# The impact of race on survival in metastatic prostate cancer: a systematic literature review

**DOI:** 10.1038/s41391-023-00710-1

**Published:** 2023-08-17

**Authors:** Stephen J. Freedland, Imtiaz A. Samjoo, Emily Rosta, Austin Lansing, Evelyn Worthington, Alexander Niyazov, Jonathan Nazari, Bhakti Arondekar

**Affiliations:** 1 Department of Urology, Cedars-Sinai Medical Center, Los Angeles, CA USA; 2 Urology Section, Durham VA Medical Center, Durham, NC USA; 3 EVERSANA™, Burlington, ON Canada; 4 Pfizer, Inc., New York, NY USA; 5 Pfizer, Inc., Collegeville, PA USA

**Keywords:** Cancer epidemiology, Outcomes research

## Abstract

**Background:**

Prostate cancer (PC) is the second most diagnosed cancer in men worldwide. While racial and ethnic differences exist in incidence and mortality, increasing data suggest outcomes by race among men with newly diagnosed PC are similar. However, outcomes among races beyond Black/White have been poorly studied. Moreover, whether outcomes differ by race among men who all have metastatic PC (mPC) is unclear. This systematic literature review (SLR) provides a comprehensive synthesis of current evidence relating race to survival in mPC.

**Methods:**

An SLR was conducted and reported in accordance with PRISMA guidelines. MEDLINE®, Embase, and Cochrane Library using the Ovid® interface were searched for real-world studies published from January 2012 to July 2022 investigating the impact of race on overall survival (OS) and prostate cancer-specific mortality (PCSM) in patients with mPC. A supplemental search of key congresses was also conducted. Studies were appraised for risk of bias.

**Results:**

Of 3228 unique records identified, 62 records (47 full-text and 15 conference abstracts), corresponding to 54 unique studies (51 United States and 3 ex-United States) reporting on race and survival were included. While most studies showed no difference between Black vs White patients for OS (*n* = 21/27) or PCSM (*n* = 8/9), most showed that Black patients demonstrated improved OS on certain mPC treatments (*n* = 7/10). Most studies found no survival difference between White patients and Hispanic (OS: *n* = 6/8; PCSM: *n* = 5/6) or American Indian/Alaskan Native (AI/AN) (OS: *n* = 2/3; PCSM: *n* = 5/5). Most studies found Asian patients had improved OS (*n* = 3/4) and PCSM (*n* = 6/6) vs White patients.

**Conclusions:**

Most studies found Black, Hispanic, and AI/AN patients with mPC had similar survival as White patients, while Black patients on certain therapies and Asian patients showed improved survival. Future studies are needed to understand what aspects of race including social determinants of health are driving these findings.

## Background

Prostate cancer is the second most common cancer among men globally, with 1.4 million new cases diagnosed in 2020 [1]. Patients with metastatic prostate cancer (mPC) have a poor prognosis, with the five-year survival rate reduced from greater than 99% in localized and regional forms of the disease to 32.3% in mPC [2]. Racial and ethnic minorities are disproportionately affected by mPC, with the greatest age-adjusted incidence rates observed in Black men [3].

Recent years have seen numerous advancements for patients with prostate cancer, including improvements in treatment, screening, and diagnosis; however, notable disparities by race and other social determinants of health (SDOH) persist in incidence, access to care, and survival [4–6]. This aligns with broader oncology trends globally, where substantial advances in cancer care have occurred, but systemic barriers often prevent these advances and their potential benefits from being fully realized in certain regions and sub-populations [7]. In response to these trends, there has been a growing interest in understanding and addressing the role of SDOH in oncology outcomes, with the American Cancer Society publishing a framework and recommendations on this topic in 2020 [8]. According to the World Health Organization, SDOH are defined as “the circumstances in which people are born, grow up, live, work, and age, as well as the systems put in place to deal with illness”, which are influenced by economic, political, and social forces [9]. Within the context of SDOH and mPC, race has been a key focus of existing literature, with other SDOH such as income, education, and geographic region recognized as key factors that can contribute to racial disparities, in addition to impacting care on their own [5, 10].

Given the growing emphasis on understanding and addressing SDOH in the oncology setting, accompanied by the poor prognosis that persists in mPC, there is a need to gain a clear picture of the current impact of SDOH – and particularly the impact of race – on survival outcomes in mPC. Although several reviews on the impact of race and other SDOH in prostate cancer have been conducted in recent years, these have been narrative in nature or have focused on prostate cancer broadly, rather than mPC [4–6, 10, 11]. Despite evidence of racial and ethnic disparities in incidence and mortality [3], increasing data suggest similar outcomes by race among men with newly diagnosed PC [3, 12]. However, to date, outcomes among races beyond Black and White have been poorly studied, and it remains unclear whether outcomes differ by race among men with mPC. Therefore, we conducted a systematic literature review (SLR) to identify and summarize evidence on the relationship between SDOH and survival, treatment access/adherence, and other clinical outcomes in patients with mPC. Given the large amount of data identified, in this article, we focus solely on findings evaluating the relationship between race and survival outcomes in patients with mPC. Our findings regarding other SDOH beyond race and other outcomes beyond survival will be reported in a future publication.

## Methods

This review was performed according to the methodology defined by the Cochrane Collaboration [13] and adhered to best practices for conduct and reporting systematic reviews, including the Preferred Reporting Items for Systematic Review and Meta-Analysis (PRISMA) statement [14, 15]. Our review protocol was designed according to PRISMA for systematic review protocols (PRISMA-P) statement and was registered with PROSPERO international prospective register of systematic reviews prior to initiating data extraction (registration number CRD42022350888). The Population, Intervention, Comparator, Outcome, Study design (PICOS) framework was used to develop the search strategy and structure the reporting of the eligibility criteria (Table A.1) [13].

### Literature Search

The search strategy was developed and executed by a medical information specialist in consultation with the review team (Table A.2). The strategy was peer-reviewed independently by another senior medical information specialist before execution using the Peer Review of Electronic Search Strategies (PRESS) checklist [16]. Searches were conducted on July 7, 2022 using the Ovid® search interface and included Embase, MEDLINE® (including Epub Ahead of Print and In-Process & Other Non-Indexed Citations, and Daily), and the Cochrane Database of Systematic Reviews. Search strategies utilized a combination of controlled vocabulary and keywords. Vocabulary and syntax were adjusted across databases. The search strategy was limited to English language and the search period spanned from 2012 onwards. This date cut-off was deemed sufficient to capture the modern era of mPC treatment based on expert clinician input. Conference abstracts were limited to those published in 2019 onwards as valuable abstracts presented prior to 2019 were presumably published by the search date. A supplementary grey literature search, which entails searching for information falling outside the mainstream of published journal articles, was conducted wherein conference abstracts were retrieved from key congresses (e.g., American Society of Clinical Oncology, European Society for Medical Oncology, and National Comprehensive Cancer Network). A full list of congresses searched is available in Table A.3.

### Study selection and data synthesis

Study selection, data extraction, and quality assessment were conducted by two independent reviewers, with discrepancies resolved by consensus or a third independent reviewer. The study selection process was performed in the DistillerSR (Evidence Partners, Ottawa, Canada) SLR software [17]. Records imported into DistillerSR underwent deduplication using the intrinsic DistillerSR deduplication algorithm. The titles and abstracts of identified citations were screened for relevance and then further evaluated in full-text form based on the pre-defined PICOS criteria (Table A.1). Data from included citations were extracted into a standardized form in Microsoft® Excel (Microsoft Corporation, Seattle, US). Publication characteristics, study setting, study methods, study participants, study findings (both quantitative and qualitative), and sources of funding were extracted. When available, multivariable results were used instead of univariable results. Additionally, clinical judgement was used, where necessary, to assess study population characteristics (such as, metastatic castration-sensitive prostate cancer [mCSPC], metastatic castration-resistant prostate cancer [mCRPC], or mixed [both mCSPC and mCRPC]) in instances that population details were not explicitly stated in the retrieved citations. Study quality was assessed using the Newcastle-Ottawa Quality Assessment Scales for cohort and case-control studies, with a maximum score of 9. The scales assess studies based on three quality or bias parameters: (1) selection of study groups, (2) comparability of groups, and (3) determination of either the exposure or outcome of interest, for case control or cohort studies, respectively [18]. Studies that received a total score of ≥7 were considered of high quality with a low risk of bias, while studies with a total score of <5 were considered of low quality, and a high risk of bias, with any score in-between considered moderate quality [19]. Only full-text publications were assessed for quality since conference abstracts often lack sufficient methodological data to assess study quality.

## Results

### Overview of included studies

**Fig. 1:**
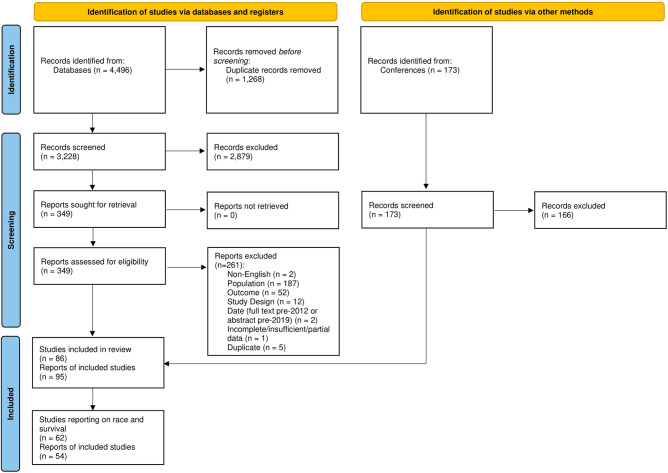
PRISMA flow diagram. Source: Page et al. 2021 [14].

Following removal of duplicates, 3228 records were identified from the database searches and screened for inclusion. A total of 173 conference abstracts were identified and screened during the supplemental search. In total, 95 records reporting on 86 unique studies met the eligibility criteria for the SLR. Of these, race and survival were reported in 62 records, corresponding to 54 unique studies (Fig. 1), and are the focus of the current paper. A full list of these 54 studies is included in Table A.4.

### Study Characteristics

The key characteristics of the included studies that assessed the impact of race on survival outcomes are summarized in Table 1. Of the 62 records, 47 were full-text publications [3, 20–65] and 15 were conference abstracts [66–80]. The 54 unique studies consisted of 53 cohort studies [3, 20–62, 64, 65, 70, 71, 73, 77–80] and 1 case control study [63]. Of these, 50 studies were retrospective [3, 20–31, 33–41, 43–63, 65, 70, 71, 73, 78–80], 3 were prospective [42, 64, 77], and 1 was mixed (prospective and retrospective) [32]. Research reported in these studies was conducted in the United States (US) (*n* = 51) [3, 20–27, 29–61, 63–65, 70, 71, 73, 77, 78, 80], Europe (*n* = 1) [62], Asia (*n* = 1) [79], and Oceania (*n* = 1) [28]. The commonly reported racial groups discussed across these studies were Black (i.e., Black, Non-Hispanic Black), White (i.e., White, Non-Hispanic White, Non-Black), Hispanic^1^, Asian (i.e., Asian, Non-Hispanic Asian, Asian/Pacific Islander), and American Indian/Alaska Native (AI/AN) (i.e., AI/AN, Native American). Twenty-seven [20, 22, 25, 27, 30, 31, 33–36, 38, 41, 48, 49, 51–58, 60, 62, 70, 71, 78] of the 54 studies reported results on overall survival (OS) or all-cause mortality, 11 studies [21–24, 26, 31, 37, 38, 44, 50, 61] reported results on prostate cancer specific mortality (PCSM), and 11 studies [29, 32, 39, 46, 62–65, 73, 77, 80] reported results on OS or PCSM in patients on specific mPC treatments, with some of the studies reporting on more than one survival outcome. Nine studies [3, 28, 40, 42, 43, 45, 47, 59, 79] reported only descriptive data, described racial groups other than Black, White, Hispanic, Asian, or Native American, such as Maori, Thai, Malaysian, or conducted separate analyses within each racial group. An overview of these studies is included in Table A.5.

**Table 1:** Summary of included studies that assessed the impact of race on survival outcomes.

Author (Year)	Type of record^a^	Study design	Retrospective or prospective	Location (Country)	mPC study population size	Relevant outcome(s)
Akinyemiju (2018) [20]	Full text	Cohort study	Retrospective	USA	10,483	OS
Bernard (2020) [21]	Full text	Cohort study	Retrospective	USA	28,979	PCSM
Borno (2020) [23]	Full text	Cohort study	Retrospective	USA	669	PCSM
Deuker (2021) [24]	Full text	Cohort study	Retrospective	USA	15,283	PCSM^b^
Garje (2020) [25]	Full text	Cohort study	Retrospective	USA	33,585	OS
He (2021) [26]	Full text	Cohort study	Retrospective	USA	47,401	PCSM
Keating (2019) [27]	Full text	Cohort study	Retrospective	USA	21,977	OS
Lao (2016) [28]	Full text	Cohort study	Retrospective	Oceania	65	Survival – See Supplement
Leuva (2019) [65]	Full text	Cohort study	Retrospective	USA	5116	OS on specific treatments
Marar (2022) [29] Marar (2021) [66] Marar (2020) [67]	Full text Conference abstract Conference abstract	Cohort study	Retrospective	USA	3808	OS on specific treatments
Parikh (2017) [30]	Full text	Cohort study	Retrospective	USA	6051	OS
Patel (2018) [31]	Full text	Cohort study	Retrospective	USA	729	OS and PCSM
Sartor (2020) [32] Sartor (2019) [68]	Full text Conference Abstract	Cohort study	Mixed	USA	1902	OS on specific treatments
Siegel (2020) [3]	Full text	Cohort study	Retrospective	USA	501,925	Survival – See Supplement
Smith (2021) [33]	Full text	Cohort study	Retrospective	USA	168	OS
Vengaloor Thomas (2020) [34] Vengaloor Thomas (2020) [69]	Full text Conference Abstract	Cohort study	Retrospective	USA	30,591	OS
Weiner (2020) [35]	Full text	Cohort study	Retrospective	USA	24,805	OS
Weiner (2021) [36]	Full text	Cohort study	Retrospective	USA	3737	OS
Wurnschimmel (2021) [37]	Full text	Cohort study	Retrospective	USA	4007	PCSM^b^
Zhang (2021) [38]	Full text	Cohort study	Retrospective	USA	24,407	OS and PCSM
Zhao (2020) [39]	Full text	Cohort study	Retrospective	USA	318	OS on specific treatments
Sheean (2022) [40]	Full text	Cohort study	Retrospective	USA	74	Survival – See Supplement
Oehrlein (2022) [41]	Full text	Cohort study	Retrospective	USA	198	OS
Hoeh (2022) [42]	Full text	Cohort study	Prospective	USA	4234	Survival – See Supplement
Hawley (2022) [64]	Full text	Cohort study	Prospective	USA	54	OS on specific treatments
Giaquinto (2022) [43]	Full text	Cohort study	Retrospective	USA	NR^c^	Survival – See Supplement
Farooq (2022) [70]	Conference Abstract	Cohort study	Retrospective	USA	51,979	OS
Wurnschimmel (2021) [44]	Full text	Cohort study	Retrospective	USA	4282	PCSM^b^
Khan (2021) [45]	Full text	Cohort study	Retrospective	USA	2008	Survival – See Supplement
Gupta (2021) [71]	Conference Abstract	Cohort study	Retrospective	USA	458	OS
George (2021) [46] Mcnamara (2019) [72]	Full text Conference Abstract	Cohort study	Retrospective	USA	2910	OS on specific treatments
Elmehrath (2021) [47]	Full text	Cohort study	Retrospective	USA	26,168	Survival – See Supplement
Zhang (2020) [73]	Conference Abstract	Cohort study	Retrospective	USA	179	OS on specific treatments
Patel (2020) [48] Patel (2020) [74]	Full text Conference Abstract	Cohort study	Retrospective	USA	837	OS
Ng (2021) [62] Ng (2020) [76] Ng (2020) [75]	Full text Conference abstract Conference abstract	Cohort study	Retrospective	Europe	425	OS and OS on specific treatments
Muralidhar (2020) [77]	Conference Abstract	Cohort study	Prospective	USA	474	OS on specific treatments
Lec (2020) [49]	Full text	Cohort study	Retrospective	USA	23,322	OS
Jogerst (2020) [78]	Conference Abstract	Cohort study	Retrospective	USA	NR^c^	OS
Deuker (2020) [50]	Full text	Cohort study	Retrospective	USA	14,125	PCSM^b^
Zhao (2019) [51]	Full text	Cohort study	Retrospective	USA	16,643	OS
Mazzone (2019) [52]	Full text	Cohort study	Retrospective	USA	529	OS
Lim (2019) [79]	Conference Abstract	Cohort study	Retrospective	Asia	92	Survival – See Supplement
Becker (2019) [53]	Full text	Cohort study	Retrospective	USA	184	OS
Guo (2018) [54]	Full text	Cohort study	Retrospective	USA	9925	OS
Ramalingam (2017) [63]	Full text	Case Control Study	Retrospective	USA	135	OS on specific treatments
Moreira (2017) [55]	Full text	Cohort study	Retrospective	USA	205	OS
Rusthoven (2016) [56]	Full text	Cohort study	Retrospective	USA	6382	OS
Schmid (2015) [57]	Full text	Cohort study	Retrospective	USA	534,011	OS
Sammon (2015) [58]	Full text	Cohort study	Retrospective	USA	100,220	OS
Muralidhar (2015) [59]	Full text	Cohort study	Retrospective	USA	41,022	Survival – See Supplement
Powell (2014) [60]	Full text	Cohort study	Retrospective	USA	13,956	OS
Taksler (2012) [61]	Full text	Cohort study	Retrospective	USA	78,927	PCSM
Bernard (2017) [22]	Full text	Cohort study	Retrospective	USA	22,293	OS and PCSM
Tutrone (2019) [80]	Conference Abstract	Cohort study	Retrospective	USA	1866	OS on specific treatments

*mPC* metastatic prostate cancer, *NR* not reported, *OS* overall survival, *PCSM* prostate cancer specific survival, *US* United States.

^a^Each row of the table represents a unique study. Records corresponding to the same study are included within the same row.

^b^Article defines outcome as cancer specific survival.

^c^Sample size of the mPC population is not reported within the article.

### Assessment of study quality

Of the 54 unique studies, 47 were full-text publications with or without abstracts and were therefore assessed for study quality using the Newcastle-Ottawa Non-Randomised Study Assessment Tool. Results of the study quality assessments are included in Table A.6 for cohort studies and Table A.7 for the one case control study. Total scores ranged from 4 to 9 out of a maximum of 9, with over 95% of studies [3, 20–63] (45 out of 47) scoring ≥7, indicating high quality and a low risk of bias [19].

### Overall survival

Overall, a total of 27 studies assessed the impact of race on OS or all-cause mortality in patients with mPC regardless of treatment received (studies that were restricted to men all receiving a single therapy or single class of therapy are discussed further below) (Table 2). Of the 27 studies, 18 reported on mCSPC populations, 6 reported on mCRPC populations, and 3 reported on mixed mCSPC/mCRPC populations. All 27 studies compared OS between Black and White patients, of which 21 (14 mCSPC, 4 mCRPC, 3 mixed) [20, 22, 25, 27, 30, 31, 33–36, 38, 41, 48, 51, 52, 55, 57, 58, 60, 62, 70] found no significant difference in OS between the two racial groups. The remaining six studies (4 mCSPC, 2 mCRPC) [49, 53, 54, 56, 71, 78] reported poorer survival in Black patients compared to White patients.

**Table 2:** OS in general, comparison by race.

Study	Population^a^	Type of analysis/results	White^b^	Black^c^	Asian^d^	Hispanic^e^	AI/AN^f^
			HR (95% CI), *p* value				
Bernard (2017) [22]	mCSPC	Log rank	Reference	*p* = 0.057	*p* < 0.001 ^g^	*p* < 0.001 ^h^	*p* = 0.398
Zhang (2021) [38]	mCSPC	Multivariable	Reference	1.02 (0.96–1.08), *p* = 0.50	0.73 (0.67-0.81), *p* < 0.0001	0.94 (0.88-1.01), *p* = 0.07	1.04 (0.81–1.32), *p* = 0.8
Weiner (2021) [36]	mCSPC	Multivariable	Reference	1.02 (0.88–1.18), *p* = 0.811	NR	0.97 (0.73–1.27), *p* = 0.809	NR
Weiner (2020) [35]	mCSPC	Multivariable	Reference	0.96 (0.91–1.00), *p* = 0.073	NR	0.78 (0.71–0.85), *p* < 0.001	NR
Vengaloor (2020) [34]	mCSPC (specifically bone metastases)	Multivariable	Reference	0.99 (0.95–1.04), *p* = NR	NR	NR	NR
	mCSPC (specifically brain, liver, or lung metastases)	Multivariable	Reference	1.00 (0.79, 1.24), *p* = NR	NR	NR	NR
Garje (2020) [25]	mCSPC	Multivariable	Reference	0.98 (0.94–1.03), *p* = 0.43	NR	NR	NR
Keating (2019) [27]	mCSPC	Multivariable	Reference	1.03 (0.98–1.07), *p* = 0.2547	NR	0.96 (0.9–1.03), *p* = 0.2601	NR
Parikh (2017) [30]	mCSPC	Multivariable	Reference	0.97 (0.88–1.06), *p* = 0.50	NR	NR	NR
Oehrlein (2022) [41]	Mixed	Multivariable	Reference	0.68 (0.24–1.95), *p* = 0.476	NR	NR	NR
Ng (2021) [62]	mCRPC	Log rank	Reference	0.81 (0.64–1.03), *p* = 0.08	NR	NR	NR
Gupta (2021) [71]	mCRPC	Multivariable	Reference	1.67 (1.05–2.65), *p* = 0.03	NR	NR	NR
Akinyemiju (2018) [20]	mCSPC	Multivariable	Reference	1.03 (0.95–1.12), *p* = NR	NR	1.00 (0.91–1.10), *p* = NR	NR
Lec (2020) [49]	mCSPC	Multivariable	Reference^i^	1.08 (1.01–1.15), *p* = NR	NR	NR	NR
Zhao (2019) [51]	mCSPC	Multivariable	Reference	1.00 (0.94–1.05), *p* = 0.901	0.72 (0.65-0.80), *p* < 0.001	NR	NR
Powell (2014) [60]	mCSPC	Univariable	Reference	*p* = 0.8399	NR	NR	NR
Moreira (2017) [55]	mCRPC	Multivariable	Reference^j^	0.85 (0.59–1.22), *p* = 0.381	NR	NR	NR
Rusthoven (2016) [56]	mCSPC	Multivariable	Reference	1.11 (1.03–1.20), *p* = 0.008	NR	NR	NR
Schmid (2015) [57]	Mixed	Multivariable	Reference	0.94 (0.87–1.01), *p* = 0.865	NR	0.93 (0.83–1.04)^k^	NR
Sammon (2015) ^l^ [58]	Mixed	Multivariable	Reference	1.04 (0.74–1.44), *p* = 0.84	NR	1.28 (0.86–1.9), *p* = 0.23	NR
Farooq (2022) [70]	mCSPC	Univariable	Reference	0.99 (0.96–1.03), *p* = 0.71	NR	NR	NR
			NR	1.048 (0.91, 1.21), *p* = 0.539	NR	NR	Reference
Jogerst (2020) [78]	mCSPC	NR	Reference	*p* < 0.001 ^m^	NR	NR	NR
Guo (2018) [54]	mCSPC	Multivariable	Reference	1.13 (1.01–1.26), *p* = 0.03	0.77 (0.40–1.48), *p* = 0.43	NR	0.73 (0.58–0.91), *p* = 0.01
Becker (2019) [53]	mCRPC	Multivariable	Reference	2.21 (1.28–3.82), *p* = NR	NR	NR	NR
Smith (2021) [33]	mCSPC	Multivariable	Reference^j^	1.11 (0.51–2.40), *p* = 0.794	NR	NR	NR
Patel (2020) [48]	mCRPC	Multivariable	Reference^j^	0.87 (0.73–1.04), *p* = 0.13	NR	NR	NR
Patel (2018) [31]	mCRPC	Multivariable	Reference^j^	0.91 (0.73–1.12), *p* = NR	NR	NR	NR
Mazzone (2019) [52]	mCSPC	Multivariable	0.75 (0.54–1.04), *p* = 0.09	Reference	NR	NR	NR

*AI/AN* American Indian and Alaska Native, *CI* confidence interval, *HR* hazard ratio, *mPC* metastatic prostate cancer, *mCRPC* metastatic castration resistant prostate cancer, *mCSPC* metastatic castration sensitive prostate cancer, *NR* not reported, *OS* overall survival.

^a^mCSPC and mCRPC population was assessed based on clinical judgement in instances that population was not explicitly stated in the text

^b^Column includes results for populations such as White, Non-Hispanic White, etc.

^c^Column includes results for populations such as Black, Non-Hispanic Black, etc.

^d^Column includes results for population such as Asian, Non-Hispanic Asian, Asian/Pacific Islander, etc.

^e^Column includes results for populations such as Hispanic, Hispanic (all races), Hispanic (White), etc.

^f^Column includes results for population such as AI/AN, Native American, etc.

^g^Asian patients had statistically significant improved OS compared to White patients.

^h^Hispanic patients had statistically significant improved OS compared to White patients.

^i^Results specifically for patients who lived longer than 2 years.

^j^Non-Black.

^k^*p* value was omitted due to inconsistent reporting in primary publication.

^l^Results shown for in-hospital mortality.

^m^Black patients have statistically significant lower OS rates compared to White patients.

Four studies [22, 38, 51, 54] that reported on Asian and White racial groups all consisted of a mCSPC population and concluded that Asian patients had improved OS compared to White patients (*n* = 3) [22, 38, 51] or found no significant differences in survival between these populations (*n* = 1) [54]. Eight studies [20, 22, 27, 35, 36, 38, 57, 58] compared OS between Hispanic and White patients. Six [20, 27, 36, 38, 57, 58] of these studies (4 mCSPC, 2 mixed) [20, 27, 36, 38, 57, 58] found no significant differences in survival between the two races; whereas, two studies [22, 35] (both with mCSPC populations) reported improved survival among Hispanic patients. Of the three studies (all mCSPC) [22, 38, 54] that compared OS between AI/AN and White patients, two [22, 38] concluded there were no significant differences in survival between the two races and one [54] reported an improved prognosis in AI/AN patients.

### Prostate cancer specific mortality

A total of 11 studies assessed the impact of race on PCSM among mPC patients regardless of treatment received (studies that were restricted to men all receiving a single therapy or single class of therapy are discussed further below) (Table 3). Nine studies [21–23, 26, 31, 37, 38, 44, 61] compared PCSM between Black and White patients, of which the majority (*n* = 8; 6 mCSPC, 1 mCRPC, 1 mixed) [22, 23, 26, 31, 37, 38, 44, 61] reported no significant differences between these two groups, while the one remaining study (mCSPC) [21] reported a worse prognosis in Black patients.

**Table 3:** PCSM in general, comparison by race.

Study	Population^a^	Type of analysis/results	White^b^	Black^c^	Asian^d^	Hispanic^e^	AI/AN^f^
			HR (95% CI), *p*-value				
Bernard (2017) [22]	mCSPC	Multivariable	Reference	1.00 (0.94–1.06), *p* = 0.982	0.81 (0.74–0.89), *p* < 0.001	0.94 (0.87–1.01), *p* = 0.094	1.23 (0.95–1.59), *p* = 0.119
Zhang (2021) [38]	mCSPC	Multivariable	Reference	1.04 (0.99–1.09), *p* = 0.10	0.77 (0.71–0.84), *p* < 0.0001	0.95 (0.89–1.00), *p* = 0.06	1.04 (0.84–1.28), *p* = 0.7
Deuker (2020)^g^[50]	mCSPC	Multivariable	Reference	NR	NR	NR	1.04 (0.80–1.36), *p* = 0.7
Bernard (2020)^h^[21]	mCSPC	Multivariable	Reference	1.12 (1.02–1.23), *p* = NR	0.70 (0.59–0.83), *p* = NR	1.00 (0.89–1.13), *p* = NR	1.27 (0.81–2.00), *p* = NR
Borno (2020) [23]	Mixed	Multivariable	Reference	1.20 (0.92–1.57), *p* = 0.1754	NR	1.20 (0.67–2.17), *p* = 0.5363	NR
Würnschimmel (2021)^g^[37]	mCSPC	Multivariable	Reference	0.93 (0.77–1.12)^i^, *p* = 0.4	0.59 (0.4–0.80)^i^, *p* < 0.0001	0.69 (0.56–0.87)^i^, *p* = 0.001	NR
Würnschimmel (2021)^g^[44]	mCSPC	Multivariable	Reference	1.01 (0.90–1.13), *p* = 0.8	0.63 (0.51–0.77), *p* < 0.001	0.97 (0.85–1.12), *p* = 0.75	NR
Deuker (2021)^g^[24]	mCSPC	Multivariable	Reference	NR	0.62 (0.57–0.69), *p* < 0.01	NR	0.86 (0.67–1.11), *p* = 0.2
Taksler (2012) [61]	mCSPC	Unclear	Reference	^j^	NR	NR	NR
Patel (2018) [31]	mCRPC	Multivariable	Reference^k^	0.94 (0.75–1.17), *p* = NR	NR	NR	NR
He (2021) [26]	Regional mCSPC	Multivariable	1.13 (0.93-1.38), *p* = 0.2089	Reference	0.74 (0.51–1.06), *p* = 0.1024	NR	1.97 (1.08–3.58), *p* = 0.0276
	Distant mCSPC	Multivariable	0.99 (0.91-1.08), *p* = 0.8534	Reference	0.66 (0.56–0.78), *p* < 0.0001	NR	0.93 (0.61–1.42), *p* = 0.7342

*AI/AN* American Indian and Alaska Native, *CI* confidence interval, *HR* hazard ratio, *mPC* metastatic prostate cancer, *mCRPC* metastatic castration resistant prostate cancer, *mCSPC* metastatic castration sensitive prostate cancer, *NR* not reported, *PCSM* prostate cancer-specific mortality.

^a^mCSPC and mCRPC population was assessed based on clinical judgement in instances that population was not explicitly stated in the text.

^b^Column includes results for populations such as White, Non-Hispanic White, etc.

^c^Column includes results for populations such as Black, Non-Hispanic Black, etc.

^d^Column includes results for population such as Asian, Non-Hispanic Asian, Asian/Pacific Islander, etc.

^e^Column includes results for populations such as Hispanic, Hispanic (all races), Hispanic (White), etc.

^f^Column includes results for population such as AI/AN, Native American, etc.

^g^Results shown for cancer-specific mortality (rather than PCSM).

^h^Results shown correspond to multivariable model that adjusted for the most complete set of covariates.

^i^97.5% CI.

^j^Study reported that “mortality did not differ significantly by [Black versus White] race for metastatic [prostate] cancers”

^k^Non-Black.

With respect to other racial groups, six studies (all mCSPC) [21, 22, 24, 37, 38, 44] compared PCSM between Asian and White patients, all of which concluded that Asian patients had significantly improved PCSM. Furthermore, six studies [21–23, 37, 38, 44] compared PCSM between Hispanic and White patients, of which five (4 mCSPC, 1 mixed) [21–23, 38, 44] found no significant difference between the two groups and one (mCSPC) [37] reported an improved prognosis in Hispanic patients. All five studies (all mCSPC) [21, 22, 24, 38, 50] comparing PCSM between AI/AN and White patients reported no significant difference between groups. Lastly, one study (mCSPC) [26] reported significantly improved PCSM in Asian patients versus Black patients with distant mPC but a worse prognosis in AI/AN patients versus Black patients with regional mPC.

### Overall survival on specific treatments

Overall, there were 11 studies which assessed the impact of race on survival while simultaneously taking into consideration the specific systemic life-prolonging mPC treatments received (Table 4), all of which were in populations of mCRPC patients. Four studies [32, 64, 77, 80] compared OS between Black and White patients treated with sipuleucel-T, and the majority (*n* = 3) [32, 77, 80] reported that OS was improved in Black patients compared to White patients, while only one study [64] found no association. Additionally, one study comparing Asian and White patients treated with sipuleucel-T found no association between race and OS [73]. One study [39] in patients treated with ^222^radium concluded that Black patients had improved OS compared to White patients. The results however were conflicting among studies where patients were treated with enzalutamide and/or abiraterone (*n* = 5; 1 reporting on abiraterone only, 1 reporting on enzalutamide only, 1 reporting on abiraterone and enzalutamide as separate cohorts, and 2 reporting on abiraterone or enzalutamide in a combined cohort) [29, 46, 62, 63, 65]. For cohorts of patients treated with abiraterone alone, one study reported improved OS in Black patients compared to White patients [29], and the other reported no difference [63]. For cohorts of patients treated with enzalutamide alone, the two identified studies found no association in OS between Black vs. White race [29, 62]. In the combined cohorts of patients treated with abiraterone or enzalutamide, both identified studies concluded that Black patients had improved OS compared to White patients [46, 65].

**Table 4:** OS on specific treatments, comparison by race.

Study	Population^a^	Type of Analysis/Results	Specific Treatment	White^b^	Black	Asian
				HR (95% CI), *p* value		
Sartor (2020) [32]	mCRPC	Multivariable, PSA matched cohort	Sipuleucel-T	Reference	0.6 (0.48–0.74), *p* < 0.001	NR
Marar (2022) [29]	mCRPC	Propensity score–based inverse probability of treatment weighting	Abiraterone	Reference	0.76 (0.60–0.98), *p*=NR	NR
			Enzalutamide	Reference	0.87 (0.66–1.14), *p*=NR	NR
Tutrone (2019) [80]	mCRPC Baseline PSA < 5	Univariable	Sipuleucel-T	Reference	0.49 (0.26–0.92), *p* = 0.026	NR
	mCRPC Baseline PSA ≥ 5	Univariable	Sipuleucel-T	Reference	0.80 (0.66–0.97), *p* = 0.024	NR
Ramalingam (2017) [63]	mCRPC	Multivariable	Abiraterone Acetate	Reference	0.678 (0.356–1.290), *p* = 0.2363	NR
Zhao (2020) [39]	mCRPC	Multivariable	^222^Radium	Reference^c^	0.75 (0.57–0.99), *p* = 0.045	NR
Hawley (2022) [64]	mCRPC	Log rank	Sipuleucel-T	Reference	*p* = 0.07	NR
George (2021) [46]	mCRPC	Multivariable	Enzalutamide or Abiraterone	Reference	0.67 (0.592–0.758), *p* < 0.0001	NR
Muralidhar (2020) [77]	mCRPC	Multivariable	Sipuleucel-T	Reference	0.542 (0.371–0.792), *p* = 0.002	NR
Leuva (2019) [65]	mCRPC	Univariable	Enzalutamide or Abiraterone	Reference	*p* = 0.02^d^	NR
Ng (2021) [62]	mCRPC	Log-rank test	Enzalutamide	Reference	*p* = 0.91	NR
Zhang (2020) [73]	mCRPC	Unclear	Sipuleucel-T	Reference	NR	0.69 (0.4–1.4), *p* = 0.2777

*CI* confidence interval, *HR* hazard ratio, *mPC* metastatic prostate cancer, *mCRPC* metastatic castration resistant prostate cancer, *mCSPC* metastatic castration sensitive prostate cancer, *NR* not reported, *OS* overall survival, *PSA* prostate specific antigen.

^a^mCSPC and mCRPC population was assessed based on clinical judgement in instances that population was not explicitly stated in the text.

^b^Column includes results for populations such as White, Non-Hispanic White, etc.

^c^Non-Black.

^d^Black patients display statistically significant improved OS compared to White patients.

## Discussion

The present review, to our knowledge, is the first to focus on race and survival outcomes specifically in patients with mPC (including mCSPC and mCRPC). Using a comprehensive and rigorous search protocol, 54 studies reported across 62 citations were identified. While nearly all the research identified was conducted in the US, a few studies were identified in other parts of the world, namely one each from Europe, Asia, and Oceania. Most of the research included in this review indicated that survival (both OS and PCSM) is not different among Black and White patients with mPC, although when receiving certain systemic life-prolonging treatments, Black patients may have improved outcomes compared to White patients. Moreover, while survival appeared to be similar between Hispanics and Whites, survival was generally better among Asians.

Interestingly, a recent meta-analysis by Vince et al. that compared outcomes between Black and White men in the US with PC (not limited to mPC) found that Black men had worse survival in studies that minimally accounted for other SDOH compared to studies that greatly accounted for other SDOH, wherein no differences by race were seen [81]. While our study was focused exclusively on mPC, we likewise found studies that showed both better or worse survival for Black men. While determining the exact degree that studies controlled for other SDOH was beyond the scope of our SLR, we did use multivariable results which commonly adjusted for factors such as age, race, stage at diagnosis, and year of diagnosis, when available. Although it is evident that Black men are disproportionately affected by mPC as observed with the high age-adjusted incidence rates [3], the multivariate results in this SLR may conceal the poor outcomes present in this minority group. It is certainly intriguing and worthy of future study to test whether the heterogeneity in results among studies in our SLR resulted from varying degrees of accounting for other SDOH.

Other recent narrative reviews however have reported disparities between Black and White patients in prostate cancer characteristics and survival outcomes [5, 10]. For instance, Lillard et al. [10] and Hinata et al. [5] focused on prostate cancer in general (i.e., not restricted to mPC) and reported that Black patients have higher mortality. The discrepancy in these recent narrative reviews compared to our results could be due to disease setting (i.e., mPC vs. non-mPC) as suggested by Hinata et al. [5], or poor accounting for known disparities in SDOH between Black and White men [81]. Hence, the absence of survival disparities between Black and White patients reported by most studies identified in the present review may in part be explained by the mPC disease setting, better standardization of treatment or access to care, and/or other variables that are directly or indirectly associated with SDOH between races. Accordingly, a large retrospective analysis of the US National Cancer Database, which was identified by the present review, supported our findings and did not observe disparities in the mPC population, but did find differences in survival between Black and White patients in the non-metastatic setting and in the setting of prostate cancer broadly (i.e., mPC and non-mPC combined) [34]. Moreover, even in a strictly non-metastatic setting, there is some evidence of similar survival outcomes between Black and White patients [12]. Hence, additional research is required to understand what aspects of race, including other SDOH, are driving survival outcomes in patients with prostate cancer.

A minority of studies in the present review did show evidence of worsened OS (*n* = 6) [49, 53, 54, 56, 71, 78] or PCSM (*n* = 1) [21] in Black patients compared to White patients. It is unclear why the results of these few studies are discordant compared to the findings of the majority of studies identified in this review. As such, it would be premature to conclude that disparities in mPC outcomes for Black and White patients have been overcome and indicates that further work in this area is still needed. While age-standardized mortality rates (ASMR) have decreased across the U.S. with a higher decrease in Black men, ASMR remain higher in this minority group [82]. Moreover, the increased rate at which Black patients are diagnosed with prostate cancer compared to White patients [3] would lead to more deaths in the Black population assuming outcomes post-diagnosis are equivalent. Thus, achieving superior survival rates or mitigating SDOH-related risk factors in Black patients may be required to ensure truly equitable treatment outcomes for these populations.

Interestingly, several studies reported improved survival in Black patients compared to White patients when taking into account specific types of systemic life-prolonging therapies such as sipuleucel-T and ^222^radium [32, 39, 77, 80]. This may be due to greater innate immune responsiveness in Black patients than White patients or differences in prostate cancer genetics, both of which may contribute to differences in outcomes on certain therapies; however, further clinical trial exploration is needed in this area [29, 32, 39]. Notably, all the identified studies in our review that reported improved survival in Black patients compared to White patients while receiving specific treatments were conducted in a mCRPC population [29, 32, 39, 46, 65, 77, 80]. This aligns with a recent narrative review that reported similar or improved response and survival outcomes in Black patients compared to White patients with mCRPC [4].

In terms of evidence for Asian patients, most identified studies reported improved survival outcomes in Asian patients versus White patients with mPC. This may be attributable to factors including genomics, diet and lifestyle, access to care, and responsiveness to treatment [22, 24, 44]. Nonetheless, regardless of these findings, prostate cancer continues to pose a substantial risk for the Asian population [83, 84]. With respect to evidence comparing survival in Hispanic and White patients with mPC, most identified studies reported no survival differences between these groups. However, it was recently noted that survival for Hispanic patients with prostate and other types of cancer varies widely across different sub-populations (e.g., Mexicans, Puerto Ricans, Cubans, and others), and that failure to account for these different subgroups may cause survival disparities to be underestimated [85]. Unfortunately, the studies included in the present review did not further sub-divide patients within the Hispanic population. Lastly, most studies comparing AI/AN versus White patients reported no difference in prognosis between these two groups, which validates findings from the one large identified study that was specifically designed to compare prognosis in Native American versus White patients and concluded that prognosis does not differ after adjusting for differences in disease stage and grade at presentation [24]. However, it should be noted that the number of studies evaluating AI/AN men were limited.

The present study made efforts to delineate study populations in terms of metastatic disease (i.e., mCSPC and mCRPC) to further identify if any racial differences exist in survival outcomes in these subgroups. The majority of studies assessing racial disparities and cancer outcomes were conducted in men with mCSPC, and most of these studies reported no significant difference in OS or PCSM between Black and White men. Similarly, of the few studies that did evaluate all mCRPC patients regardless of treatment, the majority found no such racial differences with respect to survival outcomes between Black and White men. In contrast, among more homogenous groups of patients with mCRPC all receiving the same therapy, in general OS was better in Black men treated with Sipuleucel-T, Radium-223, and half the studies of men treated with novel hormonal agents (abiraterone and/or enzalutamide). This aligns with a study by Freedland et al. which states that Black men with mCRPC treated with enzalutamide had better clinical PFS compared to White men [86]. Better survival with various treatments and yet lack of survival benefit among all men may suggest that less aggressive treatment in other studies is negating benefits for Black men, though further studies adjusting for treatment patterns are needed to confirm this. It is worthy to note that OS and PCSM outcomes between Asian vs. White patients and between AI/AN vs. White patients were only evaluated in mCSPC patients, underscoring the need for further racial disparity research in more advanced mPC.

Among the included evidence, several data gaps were identified. For instance, our review identified only three ex-US studies that evaluated race and survival in mPC and therefore, the ability to draw conclusions in other regions is limited. Additionally, the identified studies had some degree of heterogeneity with respect to the statistical tests and methodologies used (i.e., although most studies reported results from multivariable analyses, some reported only descriptive results); however, the majority of studies included in this review were sufficiently similar in methodologies/statistical tests to allow findings to be compared across studies. Moreover, heterogeneity exists in the way that race was determined across the studies [87] (i.e., self-identified, collected retrospectively from databases such as the National Cancer Database) which may account for disparities in mPC survival. Factors such as immigration and ethnic history may influence race determination, which in turn affects treatment patterns and effectiveness. For instance, Posielski et al. stated that AA men were more probable to receive radiation and less probable to receive active surveillance compared to non-AA men for localized prostate cancer [88]. As such, we acknowledge that race is a social construct, but nonetheless these results highlight important associations. Furthermore, although the majority of included studies conducted multivariable analysis, accounting for a variety of sociodemographic and disease characteristics, there remined heterogeneity across studies in the adjustment of specific social factors, limiting our ability to truly compare results across studies. Also, several studies included in this review had small sample sizes and may have been underpowered to detect modest differences between racial groups. Additionally, compared to the amount of evidence identified overall, relatively few studies assessed racial differences with respect to survival on specific treatments. Lastly, we acknowledge that two studies [64, 65] were deemed as a potential high risk of bias during quality assessment; however, including these two studies did not change the overall findings of the review, which consisted of more than 95% high-quality studies.

A key strength of the present study is that it was conducted and reported in accordance with standards for systematic reviews as outlined by Cochrane [13] and PRISMA Guidelines [14, 15], which results in a more comprehensive and rigorous assessment of the available evidence than a narrative review. This literature review also provides a more unbiased view of the evidence as studies were systematically assessed for eligibility and reasons for exclusion were documented during full-text screening. Additionally, by focusing specifically on mPC as the target population and further delineating mCSPC and mCRPC subgroups, the present SLR provides a more precise body of evidence than previous reviews that assessed racial disparities in prostate cancer broadly, thereby allowing us to identify data gaps and unmet need specifically in mPC. Moreover, we recognized that certain racial and ethnic minorities are often underrepresented in clinical trial populations, and hence we designed the SLR to focus on real-world evidence to accurately capture the current impact of race on survival outcomes in mPC. We included full-text articles published in past 10 years to capture the growing interest in SDOH, especially race, over the past decade. Interestingly, over 77%, or 48 of the 62 studies, were published within the last four years, suggesting significantly more growth in the past few years compared to the earlier half of the decade, particularly in the US.

As with any study, the present research was not without limitations. For instance, the literature search was limited to English language only, meaning that findings from non-English publications are not reflected in the study conclusions. Also, in our comparison of survival outcomes in White versus Black patients, studies that reported “non-Black” populations were considered together with those reporting “White” populations. Although this could have resulted in some study participants being misclassified, this is likely a minor limitation as the non-Black populations were considered in only five studies [31, 33, 39, 48, 55] and consisted of 90–99% White patients, when reported. Next, the present review included conference abstracts, which often have limited data or information; however, the inclusion of abstracts allowed the most recent evidence in the field to be incorporated. Additionally, given that the present review was qualitative, there remains a need for future studies that quantitatively explore the effects of race and survival in a meta-analysis. Lastly, the prognosis of patients with mPC is likely influenced by SDOH apart from race, indicating that future studies exploring the impact of other SDOH in mPC are required. Accordingly, findings related to other SDOH and outcomes included in this SLR will be reported in a future publication.

In conclusion, this study provided a comprehensive, contemporary, systematic assessment of the impact of race on survival in the mPC population. Findings suggest that disparities between Black and White patients with mPC may not be as pronounced as those observed in prostate cancer more broadly. In contrast, Asian patients often display improved survival outcomes compared to White patients with mPC. Most evidence showed no difference in survival between White patients and AI/AN or Hispanic patients with mPC, respectively, though future studies need to examine specific Hispanic subpopulations. Further work is needed to understand the impact of race on survival across the spectrum of prostate cancer (e.g., early, advanced, metastatic, castration sensitive, castration resistant, etc.). It is hoped that this review can provide new directions for research in understanding, as well as addressing, social determinants of health.

## Supplementary information

Supplementary Appendix

## Footnotes

1 The Hispanic category includes results for populations such as Hispanic, Hispanic (all races), Hispanic (White), etc. as some studies consider Hispanic an ethnicity and differentiate included patients with reference to race.
