# Supplementary material for: The impact of race on survival in metastatic prostate cancer: a systematic literature review
Source: Prostate Cancer Prostatic Dis. 2023 Aug 17;26(3):461–74. doi: 10.1038/s41391-023-00710-1 (PMC10449629; doi:10.1038/s41391-023-00710-1)
Supplement: Supplementary file 1 — Supplementary Appendix [file 41391_2023_710_MOESM1_ESM.docx]

The Impact of Race on Survival in Metastatic Prostate Cancer:
A Systematic Literature Review

**Supplemental Appendix**

**Table A.1: PICOS Criteria**

|  | Inclusion Criteria | Exclusion Criteria | |
| --- | --- | --- | --- |
| *Population* | Study populations or subgroups of patients (humans only; men) with:   - Age ≥18 years - Histologically or cytologically confirmed adenocarcinoma of the prostate - Metastatic disease - Castration-sensitive OR castration-resistant | Study populations or subgroups:   - Non-human - Age <18 years - Non-metastatic disease | |
| *Interventions/ Comparators* | Any not excluded | Alternative medicines | |
| *Outcomes* | Studies will be identified that mention a relationship between at least one SDOH (see following page) and target outcome:  **Clinical outcomes of interest:**   - Real-world progression-free survival - Overall survival - Objective response rate - Treatment duration - Real-world patient-reported outcomes - Time to subsequent therapy/chemotherapy   **Adherence/access outcomes of interest:**   - Medication access (payer rejection rates, etc) - Medication persistence (abandonment rates, etc) - Medication adherence - Treatment intensity/intensification - Initiation of life-prolonging therapies - Treatment consistent with guidelines - Health insurance access | Screening for prostate cancer  Epidemiology | |
| *Study Design* | Real-world data | Clinical trials  Systematic literature reviews  Animal-only studies  Opinion pieces  Letters  Narrative reviews  Case studies  Case control studies comparing mPC patients to non-mPC patients | |
| *Language* | Articles in English | All non-English articles | |
| *Dates* | Database search (full texts): 2012 to present  Database search (abstracts): 2019 to present | | Database search (full texts): full texts published 2011 or earlier  Database search (abstracts): abstracts published 2018 or earlier |

Table A.2: Search Strategy

| **#** | **Searches** | **Results** |
| --- | --- | --- |
| 1 | exp Prostatic Neoplasms/sc | 823 |
| 2 | exp Prostatic Neoplasms/ or (((prostate or prostatic) adj3 (adenocarcinoma$ or adeno-carcinoma$ or cancer$ or carcinoma$ or malignan$ or neoplas$ or tumor? or tumour?)) or ((PC or PCa or mPC) and (prostate or prostatic))).ti,ab,kf,kw. [Prostate Cancer] | 492619 |
| 3 | exp Neoplasm Metastasis/ or (metasta$ or ((disseminat$ or spread$ or secondary or migrat$ or seeding) adj3 (neoplas$ or cancer$ or carcinoma$ or adenocarcinoma$ or adeno-carcinoma$ or tumo?r? or sarcoma)) or micrometasta$ or advanced or incurable or late stage? or lethal$ or noncurable or non-curable or progressive or stage IV or terminal or uncurable).ti,ab,kf,kw. [Metastatic Terms] | 4809095 |
| 4 | 1 or (2 and 3) [Metastatic Prostate Cancer] | 156272 |
| 5 | exp Socioeconomic Factors/ or Social Determinants of Health/ or exp Health Literacy/ or Social Support/ or ((health adj3 social adj3 determinant?) or (socioeconomic$ adj3 factor?) or ((living or social) adj3 (standard? or condition?)) or inequalit$ or (medical adj2 indigenc$) or ((economic or educational or employ$ or occupation$) adj3 (status$ or achievement?)) or (academic adj2 failure?) or literacy or illiteracy or (employment adj3 (termination or precarious or marginal or insecurit$)) or underemployment or unemployment or (labo?r adj2 force?) or income? or low-income? or poverty or indigent? or (health adj2 literacy) or ((social or psychosocial or psychological) adj2 support?)).ti,ab,kf,kw. [SDOH Terms] | 1587033 |
| 6 | Health Services Accessibility/ or Healthcare Disparities/ or "Health Services Needs and Demand"/ or "Treatment Adherence and Compliance"/ or Population Health Management/ or Minority Health/ or ((((health adj3 (care or service$)) or healthcare) adj3 (disparit$ or inequality$ or equit$ or right? or barrier? or availabilit$ or access$ or delivery or need?)) or (medically adj3 underserved adj3 (area? or population? or people? or person?)) or ((therap$ or treatment? or medication? or drug? or medicine? or program? or service$) adj4 (availabilit$ or access$)) or ((therap$ or treatment? or drug? or medication? or medicine?) adj4 ((adhere$ and complian$) or persisten$)) or ((population? or group? or communit$ or public$2) adj3 health adj3 manage$) or minorit$ health).ti,ab,kf,kw. [Healthcare Disparities & Access] | 879785 |
| 7 | Race Factors/ or Race Relations/ or exp Racism/ or exp Racial Groups/ or exp Ethnicity/ or Vulnerable Populations/ or (((race? or ethnic$ or racial$) adj3 (factor? or relation? or prejudice? or bias$ or discrimination? or group? or stock? or segregation or disparit$3 or equality or equity or inequit$3)) or (racist adj3 (behavio?r? or prejudice? or bias$)) or (racism? adj3 (covert or everyday or systemic or institutional$ or structural)) or interracial relation? or social discrimination or ethnicism or ((american? or canadian? or alaskan?) adj2 (native? or indigenous)) or african american? or asian? or asiatic? or black? or negro$ or caucasian? or hispanic? or indian? or amerind? or latino? or latina? or first nation? or metis or inuit? or kalaallit? or inupiat? or aleut? or eskimo? or pacific islander? or white? or caucasian? or caucasoid? or "people of color" or "people of colour" or diverse population? or environmental justice or ethnic group? or ethnic population? or minority group? or minority population? or ghetto? or slum? or ((vulnerable or underserved or disadvantaged or sensitive) adj3 (population? or patient?))).ti,ab,kf,kw. [Race & Ethnic Groups & Racism] | 2335805 |
| 8 | 5 or 6 or 7 | 4353374 |
| 9 | 4 and 8 | 7778 |
| 10 | (address or autobiography or bibliography or biography or comment or dictionary or directory or editorial or "expression of concern" or festschrift or historical article or interactive tutorial or lecture or legal case or legislation or news or newspaper article or patient education handout or personal narrative or portrait or video-audio media or webcast or (letter not (letter and randomized controlled trial))).pt. | 4683658 |
| 11 | exp Animals/ not (exp Animals/ and Humans/) [ANIMAL-ONLY REMOVED] | 17090348 |
| 12 | 10 or 11 | 21221882 |
| 13 | 9 not 12 [Remove Animal studies and Opinion pieces] | 5672 |
| 14 | 13 use ppez [MEDLINE results] | 2206 |
| 15 | exp prostate tumor/ or (((prostate or prostatic) adj3 (adenocarcinoma$ or adeno-carcinoma$ or cancer$ or carcinoma$ or malignan$ or neoplas$ or tumor? or tumour?)) or ((PC or PCa or mPC) and (prostate or prostatic))).ti,ab,kf,kw. [PROSTATE CANCER] | 470259 |
| 16 | exp metastasis/ or (metasta$ or ((disseminat$ or spread$ or secondary or migrat$ or seeding) adj3 (neoplas$ or cancer$ or carcinoma$ or adenocarcinoma$ or adeno-carcinoma$ or tumo?r? or sarcoma)) or micrometasta$ or advanced or incurable or late stage? or lethal$ or noncurable or non-curable or progressive or stage IV or terminal or uncurable).ti,ab,kf,kw. [METASTATIC TERMS] | 4809095 |
| 17 | 15 and 16 [Metastatic Prostate Cancer] | 153362 |
| 18 | exp socioeconomics/ or "social determinants of health"/ or exp health literacy/ or social support/ or ((health adj3 social adj3 determinant?) or (socioeconomic$ adj3 factor?) or ((living or social) adj3 (standard? or condition?)) or inequalit$ or (medical adj2 indigenc$) or ((economic or educational or employ$ or occupation$) adj3 (status$ or achievement?)) or (academic adj2 failure?) or literacy or illiteracy or (employment adj3 (termination or precarious or marginal or insecurit$)) or underemployment or unemployment or (labo?r adj2 force?) or income? or low-income? or poverty or indigent? or (health adj2 literacy) or ((social or psychosocial or psychological) adj2 support?)).ti,ab,kf,kw. [SDOH Terms] | 1235345 |
| 19 | exp health care access/ or healthcare disparity/ or health service/ or exp patient compliance/ or population health management/ or minority health/ or ((((health adj3 (care or service$)) or healthcare) adj3 (disparit$ or inequality$ or equit$ or right? or barrier? or availabilit$ or access$ or delivery or need?)) or (medically adj3 underserved adj3 (area? or population? or people? or person?)) or ((therap$ or treatment? or medication? or drug? or medicine? or program? or service$) adj4 (availabilit$ or access$)) or ((therap$ or treatment? or drug? or medication? or medicine?) adj4 ((adhere$ and complian$) or persisten$)) or ((population? or group? or communit$ or public$2) adj3 health adj3 manage$) or minorit$ health).ti,ab,kf,kw. [Healthcare Disparities & Access] | 951135 |
| 20 | race/ or exp race relation/ or exp ancestry group/ or exp ethnic/ or racial aspects/ or vulnerable population/ or (((race? or ethnic$ or racial$) adj3 (factor? or relation? or prejudice? or bias$ or discrimination? or group? or stock? or segregation or disparit$3 or equality or equity or inequit$3)) or (racist adj3 (behavio?r? or prejudice? or bias$)) or (racism? adj3 (covert or everyday or systemic or institutional$ or structural)) or interracial relation? or social discrimination or ethnicism or ((american? or canadian? or alaskan?) adj2 (native? or indigenous)) or african american? or asian? or asiatic? or black? or negro$ or caucasian? or hispanic? or indian? or amerind? or latino? or latina? or first nation? or metis or inuit? or kalaallit? or inupiat? or aleut? or eskimo? or pacific islander? or white? or caucasian? or caucasoid? or "people of color" or "people of colour" or diverse population? or environmental justice or ethnic group? or ethnic population? or minority group? or minority population? or ghetto? or slum? or ((vulnerable or underserved or disadvantaged or sensitive) adj3 (population? or patient?))).ti,ab,kf,kw. [Race & Ethnic Groups & Racism] | 2262235 |
| 21 | 18 or 19 or 20 | 4044577 |
| 22 | 17 and 21 | 7862 |
| 23 | (comment or editorial or news or newspaper article or (letter not (letter and randomized controlled trial))).pt. [OPINION PIECES REMOVE - Embase] | 4244868 |
| 24 | (exp animal/ or exp animal experimentation/ or exp animal model/ or exp animal experiment/ or nonhuman/ or exp vertebrate/) not (exp human/ or exp human experimentation/ or exp human experiment/) [ANIMAL STUDIES ONLY - REMOVE - EMBASE] | 11980080 |
| 25 | 23 or 24 | 16070648 |
| 26 | 22 not 25 [Remove Animal studies and Opinion pieces] | 7712 |
| 27 | conference abstract.pt. | 4443410 |
| 28 | 26 not 27 [CONFERENCE ABSTRACTS REMOVED] | 5310 |
| 29 | 26 and 27 | 2402 |
| 30 | limit 29 to yr="2019 -Current" | 873 |
| 31 | 28 or 30 [MOST RECENT 3 YRS CONFERENCE ABSTRACTS RETAINED] | 6183 |
| 32 | 31 use oemezd [Embase results] | 4142 |
| 33 | (((prostate or prostatic) adj3 (adenocarcinoma$ or adeno-carcinoma$ or cancer$ or carcinoma$ or malignan$ or neoplas$ or tumor? or tumour?)) or ((PC or PCa or mPC) and (prostate or prostatic))).ti,ab,kw. | 404852 |
| 34 | (metasta$ or ((disseminat$ or spread$ or secondary or migrat$ or seeding) adj3 (neoplas$ or cancer$ or carcinoma$ or adenocarcinoma$ or adeno-carcinoma$ or tumo?r? or sarcoma)) or micrometasta$ or advanced or incurable or late stage? or lethal$ or noncurable or non-curable or progressive or stage IV or terminal or uncurable).ti,ab,kw. | 4579751 |
| 35 | 33 and 34 [Metastatic Prostate Cancer] | 130850 |
| 36 | ((health adj3 social adj3 determinant?) or (socioeconomic$ adj3 factor?) or ((living or social) adj3 (standard? or condition?)) or inequalit$ or (medical adj2 indigenc$) or ((economic or educational or employ$ or occupation$) adj3 (status$ or achievement?)) or (academic adj2 failure?) or literacy or illiteracy or (employment adj3 (termination or precarious or marginal or insecurit$)) or underemployment or unemployment or (labo?r adj2 force?) or income? or low-income? or poverty or indigent? or (health adj2 literacy) or ((social or psychosocial or psychological) adj2 support?)).ti,ab,kw. [SDOH Terms] | 809526 |
| 37 | ((((health adj3 (care or service$)) or healthcare) adj3 (disparit$ or inequality$ or equit$ or right? or barrier? or availabilit$ or access$ or delivery or need?)) or (medically adj3 underserved adj3 (area? or population? or people? or person?)) or ((therap$ or treatment? or medication? or drug? or medicine? or program? or service$) adj4 (availabilit$ or access$)) or ((therap$ or treatment? or drug? or medication? or medicine?) adj4 ((adhere$ and complian$) or persisten$)) or ((population? or group? or communit$ or public$2) adj3 health adj3 manage$) or minorit$ health).ti,ab,kw. [Healthcare Disparities & Access] | 429708 |
| 38 | (((race? or ethnic$ or racial$) adj3 (factor? or relation? or prejudice? or bias$ or discrimination? or group? or stock? or segregation or disparit$3 or equality or equity or inequit$3)) or (racist adj3 (behavio?r? or prejudice? or bias$)) or (racism? adj3 (covert or everyday or systemic or institutional$ or structural)) or interracial relation? or social discrimination or ethnicism or ((american? or canadian? or alaskan?) adj2 (native? or indigenous)) or african american? or asian? or asiatic? or black? or negro$ or caucasian? or hispanic? or indian? or amerind? or latino? or latina? or first nation? or metis or inuit? or kalaallit? or inupiat? or aleut? or eskimo? or pacific islander? or white? or caucasian? or caucasoid? or "people of color" or "people of colour" or diverse population? or environmental justice or ethnic group? or ethnic population? or minority group? or minority population? or ghetto? or slum? or ((vulnerable or underserved or disadvantaged or sensitive) adj3 (population? or patient?))).ti,ab,kw. [Race & Ethnic Groups & Racism] | 2066501 |
| 39 | 36 or 37 or 38 | 3090456 |
| 40 | 35 and 39 | 6000 |
| 41 | address or autobiography or bibliography or biography or comment or dictionary or directory or editorial or "expression of concern" or festschrift or historical article or interactive tutorial or lecture or legal case or legislation or news or newspaper article or patient education handout or personal narrative or portrait or video-audio media or webcast or (letter not (letter and randomized controlled trial))).pt. | 4683658 |
| 42 | 40 not 41 [Remove Opinion pieces] | 5976 |
| 43 | 42 use coch [CDSR results] | 1 |
| 44 | 14 or 32 or 43 [ALL DATABASE RESULTS] | 6349 |
| 45 | limit 44 to yr="2012-current" | 4496 |
| 46 | remove duplicates from 45 [TOTAL UNIQUE RECORDS] | 3264^a^ |
| 47 | 46 use ppez [MEDLINE UNIQUE RECORDS] | 1417 |
| 48 | 46 use oemezd [EMBASE UNIQUE RECORDS] | 1846 |
| 49 | 46 use coch [CDSR UNIQUE RECORDS] | 1 |

^a^ An additional 36 duplicates were removed using DistillerSR.

Table A.3: List of Congresses Manually Searched

| **Congress** |
| --- |
| American Society of Clinical Oncology |
| Academy of Managed Care Pharmacy |
| Academy of Managed Care Pharmacy – Nexus |
| American Association for Cancer Research |
| American Society of Clinical Oncology – Genitourinary Cancers Symposium |
| American Society of Clinical Oncology – Quality |
| American Urological Association |
| EAU Section of Oncological Urology |
| European Association of Urology |
| European CanCer Organization |
| European Multidisciplinary Congress on Urological Cancers |
| European Society for Medical Oncology |
| International Society for Pharmacoeconomics and Outcomes Research – EU |
| International Society for Pharmacoeconomics and Outcomes Research – US |
| National Comprehensive Cancer Network |
| Society of Urologic Oncology |
| American Society of Clinical Oncology |

Table A.4: List of Included Studies Reporting on Race and Survival

| **Study Number** | **Citation of Associated Record** | **Type of Record** |
| --- | --- | --- |
| 1 | Sartor, O;Armstrong, AJ;Ahaghotu, C;McLeod, DG;Cooperberg, MR;Penson, DF;Kantoff, PW;Vogelzang, NJ;Hussain, A;Pieczonka, CM;Shore, ND;Quinn, DI;Small, EJ;Heath, EI;Tutrone, RF;Schellhammer, PF;Harmon, M;Chang, NN;Sheikh, NA;Brown, B;Freedland, SJ;Higano, CS (2020). Survival of African-American and Caucasian men after sipuleucel-T immunotherapy: outcomes from the PROCEED registry. Prostate cancer and prostatic diseases, 23(3), 517-526. | Full text |
|  | Sartor, A. O., Armstrong, A. J., Ahaghotu, C., McLeod, D. G., Cooperberg, M. R., Penson, D. F., Kantoff, P. W., Vogelzang, N. J., Hussain, A., Pieczonka, C. M., Shore, N. D., Quinn, D. I., Small, E. J., Heath, E. I., Tutrone, R. F., Schellhammer, P. F., Harmon, M., Chang, N. N., Freedland, S. J., Higano, C. S. (2019). Overall survival (OS) of African-American (AA) and Caucasian (CAU) men who received sipuleucel-T for metastatic castration-resistant prostate cancer (mCRPC): Final PROCEED analysis Journal of Clinical Oncology, 37(Supplement 15) | Conference abstract/poster |
| 2 | Vengaloor Thomas, T;Gordy, XZ;Lirette, ST;Albert, AA;Gordy, DP;Vijayakumar, S;Vijayakumar, V (2020). Lack of racial survival differences in metastatic prostate cancer in National Cancer Data Base (NCDB): a different finding compared to non-metastatic disease. Frontiers in Oncology, 10, 533070. | Full text |
|  | Vengaloor Thomas, T., Gordy, X., Lirette, S. T., Albert, A. A., Gordy, D., Vijayakumar, S., Vijayakumar, V. (2020). The Lack of Racial Survival Differences in Metastatic Prostate Cancer in National Cancer Data Base (NCDB): A Paradox Compared to Non-metastatic Disease International Journal of Radiation Oncology Biology Physics, 108(3 Supplement), e410-e411 | Conference abstract/poster |
| 3 | George, D. J., Ramaswamy, K., Huang, A., Russell, D., Mardekian, J., Schultz, N. M., Janjan, N., Freedland, S. J. (2021). Survival by race in men with chemotherapy-naive enzalutamide- or abiraterone-treated metastatic castration-resistant prostate cancer Prostate Cancer and Prostatic Diseases, 25(3):524-530 | Full text |
|  | McNamara, M. A., George, D. J., Ramaswamy, K., Lechpammer, S., Mardekian, J., Schultz, N. M., Wang, L., Baser, O., Huang, A., & Freedland, S. J. (2019). Overall survival by race in chemotherapy-naïve metastatic castration-resistant prostate cancer (mcrpc) patients treated with abiraterone acetate or enzalutamide. Journal of Clinical Oncology, 37(7_suppl), 212–212. https://doi.org/10.1200/jco.2019.37.7_suppl.212 | Conference abstract/poster |
| 4 | Patel, D. N., Howard, L. E., De Hoedt, A. M., Amling, C. L., Aronson, W. J., Cooperberg, M. R., Kane, C. J., Klaassen, Z. W., Terris, M. K., Freedland, S. J. (2020). Race does not predict skeletal-related events and all-cause mortality in men with castration-resistant prostate cancer Cancer, 126(14), 3274-3280 | Full text |
|  | Patel, D., Howard, L., Amling, C., Aronson, W., Klaassen, Z., Terris, M., Kane, C., Freedland, S. (2020). Race does not predict skeletal related events and all-cause mortality in men with castrate resistant prostate cancer Journal of Urology, 203(Supplement 4), e367 | Conference abstract/poster |
| 5 | Ng, K., Wilson, P., Mutsvangwa, K., Shamash, J. (2020). Overall survival of black and white men with metastatic castrate-resistant prostate cancer: A retrospective analysis across 20 years in the largest healthcare trust in the United Kingdom Journal of Clinical Oncology, 38(6 Supplement) | Conference abstract/poster |
|  | Ng, K., Wilson, P., Mutsvangwa, K., Alifrangis, C., Shamash, J. (2020). Overall Survival of Black and White Men with Metastatic Castration-resistant Prostate Cancer: a 20-year Retrospective Analysis from an Ethnically Diverse Area Clinical Oncology, 32(5), e130 | Conference abstract/poster |
|  | Ng, K;Wilson, P;Mutsvangwa, K;Hounsome, L;Shamash, J (2021). Overall survival of black and white men with metastatic castration-resistant prostate cancer (mCRPC): a 20-year retrospective analysis in the largest healthcare trust in England. Prostate Cancer and Prostatic Diseases, 24(3), 718-724. | Full text |
| 6 | Marar, M., Mamtani, R., Narayan, V., Vapiwala, N., Parikh, R. B. (2020). Racial disparities in utilization and effectiveness of first-line therapies in metastatic castrate resistant prostate cancer Journal of Clinical Oncology, 38(Suppl 15) | Conference abstract/poster |
|  | Marar, M;Long, Q;Mamtani, R;Narayan, V;Vapiwala, N;Parikh, RB (2022). Outcomes Among African American and Non-Hispanic White Men With Metastatic Castration-Resistant Prostate Cancer With First-Line Abiraterone. JAMA network open, 5(1), e2142093-e2142093. | Full text |
|  | Marar, M., Long, Q., Mamtani, R., Narayan, V., Vapiwala, N., Parikh, R. B. (2021). Racial disparities in efficacy of first-line abiraterone in metastatic castrate-resistant prostate cancer (mCRPC) Journal of Clinical Oncology, 39(6 SUPPL) | Conference abstract/poster |
| 7 | Lec, P. M., Lenis, A. T., Brisbane, W., Sharma, V., Golla, V., Gollapudi, K., Blumberg, J., Drakaki, A., Bergman, J., Chamie, K. (2020). Trends in palliative care interventions among patients with advanced bladder, prostate, or kidney cancer: A retrospective cohort study Urologic Oncology: Seminars and Original Investigations, 38(11), 854 | Full text |
| 8 | Akinyemiju, T;Sakhuja, S;Waterbor, J;Pisu, M;Altekruse, SF (2018). Racial/ethnic disparities in de novo metastases sites and survival outcomes for patients with primary breast, colorectal, and prostate cancer. Cancer Medicine, 7(4), 1183-1193. | Full text |
| 9 | Bernard, B;Burnett, C;Sweeney, CJ;Rider, JR;Sridhar, SS (2020). Impact of age at diagnosis of de novo metastatic prostate cancer on survival. Cancer, 126(5), 986-993. | Full text |
| 10 | Bernard, B;Muralidhar, V;Chen, Y-H;Sridhar, SS;Mitchell, EP;Pettaway, CA;Carducci, MA;Nguyen, PL;Sweeney, CJ (2017). Impact of ethnicity on the outcome of men with metastatic, hormone‐sensitive prostate cancer. Cancer, 123(9), 1536-1544. | Full text |
| 11 | Borno, HT;Cowan, JE;Zhao, S;Broering, JM;Carroll, PR;Ryan, CJ (2020, October). Examining initial treatment and survival among men with metastatic prostate cancer: An analysis from the CaPSURE registry. In Urologic Oncology: Seminars and Original Investigations (Vol. 38, No. 10, pp. 793-e1). Elsevier. | Full text |
| 12 | Deuker, M;Stolzenbach, LF;Pecoraro, A;Rosiello, G;Luzzago, S;Tian, Z;Saad, F;Chun, FKH;Karakiewicz, PI (2021). PSA, stage, grade and prostate cancer specific mortality in Asian American patients relative to Caucasians according to the United States Census Bureau race definitions. World Journal of Urology, 39(3), 787-796. | Full text |
| 13 | Garje, R;Chennamadhavuni, A;Mott, SL;Chambers, IM;Gellhaus, P;Zakharia, Y;Brown, JA (2020). Utilization and outcomes of surgical castration in comparison to medical castration in metastatic prostate cancer. Clinical genitourinary cancer, 18(2), e157-e166. | Full text |
| 14 | He, H;Han, D;Xu, F;Lyu, J (2021). How socioeconomic and clinical factors impact prostate‐cancer‐specific and other‐cause mortality in prostate cancer stratified by clinical stage: Competing‐risk analysis. The Prostate, 82(4), 415-424. | Full text |
| 15 | Keating, MJ;Giscombe, L;Tannous, T;Reddy, N;Mukkamalla, SKR;DeSouza, A;Rathore, R (2019). Age-dependent overall survival benefit of androgen deprivation therapy for metastatic prostate cancer. Journal of Oncology Pharmacy Practice, 25(8), 1927-1932. | Full text |
| 16 | Lao, C;Obertova, Z;Brown, C;Scott, N;Edlin, R;Gilling, P;Holmes, M;Tyrie, L;Lawrenson, R (2016). Differences in survival between Māori and New Zealand Europeans with prostate cancer. European Journal of Cancer Care, 25(2), 262-268 | Full text |
| 17 | Leuva, H;Sigel, K;Zhou, M;Wilkerson, J;Aggen, DH;Park, Y-HA;Anderson, CB;Hsu, T-CM;Langhoff, E;McWilliams, G;Drake, CG;Simon, R;Bates, SE;Fojo, T (2019, August). A novel approach to assess real-world efficacy of cancer therapy in metastatic prostate cancer. Analysis of national data on Veterans treated with abiraterone and enzalutamide. In Seminars in Oncology (Vol. 46, No. 4-5, pp. 351-361). WB Saunders. | Full text |
| 18 | Parikh, RR;Byun, J;Goyal, S;Kim, IY (2017). Local therapy improves overall survival in patients with newly diagnosed metastatic prostate cancer. The Prostate, 77(6), 559-572. | Full text |
| 19 | Patel, DN;Jha, S;Howard, LE;Amling, CL;Aronson, WJ;Cooperberg, MR;Kane, CJ;Terris, MK;Chapin, BF;Freedland, SJ (2018). Impact of prior local therapy on overall survival in men with metastatic castration‐resistant prostate cancer: results from Shared Equal Access Regional Cancer Hospital. International Journal of Urology, 25(12), 998-1004. | Full text |
| 20 | Siegel, DA;O'Neil, ME;Richards, TB;Dowling, NF;Weir, HK (2020). Prostate cancer incidence and survival, by stage and race/ethnicity—United States, 2001–2017. Morbidity and Mortality Weekly Report, 69(41), 1473. | Full text |
| 21 | Smith, KER;Brown, JT;Wan, L;Liu, Y;Russler, G;Yantorni, L;Caulfield, S;Lafollette, J;Moore, M;Kucuk, O;Carthon, B;Nazha, B;Bilen, MA (2021). Clinical outcomes and racial disparities in metastatic hormone-sensitive prostate cancer in the era of novel treatment options. The oncologist, 26(11), 956-964. | Full text |
| 22 | Weiner, AB;Cohen, JE;DeLancey, JO;Schaeffer, EM;Auffenberg, GB (2020). Surgical versus medical castration for metastatic prostate cancer: use and overall survival in a national cohort. The Journal of urology, 203(5), 933-939. | Full text |
| 23 | Weiner, AB;Ko, OS;Li, EV;Vo, AX;Desai, AS;Breen, KJ;Nadler, RB;Morgans, AK (2021). Survival following upfront chemotherapy for treatment-naïve metastatic prostate cancer: a real-world retrospective cohort study. Prostate Cancer and Prostatic Diseases, 24(1), 261-267. | Full text |
| 24 | Wurnschimmel, C;Wenzel, M;Colla Ruvolo, C;Nocera, L;Tian, Z;Saad, F;Briganti, A;Shariat, SF;Mirone, V;Chun, FK;Tilki, D;Graefen, M;Karakiewicz, PI (2021). Life expectancy in metastatic prostate cancer patients according to racial/ethnic groups. International Journal of Urology, 28(8), 862-869. | Full text |
| 25 | Zhang, AC;Rasul, R;Golden, A;Feuerstein, MA (2021). Incidence and mortality trends of metastatic prostate cancer: Surveillance, Epidemiology, and End Results database analysis. Canadian Urological Association Journal, 15(12), E637. | Full text |
| 26 | Zhao, H;Howard, LE;De Hoedt, A;Terris, MK;Amling, CL;Kane, CJ;Cooperberg, MR;Aronson, WJ;Klaassen, Z;Polascik, TJ;Vidal, AC;Freedland, SJ (2020). Racial discrepancies in overall survival among men treated with 223radium. The Journal of urology, 203(2), 331-337. | Full text |
| 27 | Sheean, P. M., O'Connor, P., Joyce, C., Vasilopoulos, V., Badami, A., Stolley, M. (2022). Clinical Features and Body Composition in Men with Hormone-Sensitive Metastatic Prostate Cancer: A Pilot Study Examining Differences by Race Prostate Cancer, 2022, 9242243 | Full text |
| 28 | Oehrlein, N., Streicher, S. A., Kuo, H. C., Chaurasia, A., McFadden, J., Nousome, D., Chen, Y., Stroup, S. P., Musser, J., Brand, T., Porter, C., Rosner, I. L., Chesnut, G. T., Onofaro, K. C., Rebbeck, T. R., D'Amico, A., Lu-Yao, G., Cullen, J. (2022). Race-specific prostate cancer outcomes in a cohort of military health care beneficiaries undergoing surgery: 1990-2017 Cancer Medicine, doi: 10.1002/cam4.4787. Online ahead of print. | Full text |
| 29 | Hoeh, B., Wurnschimmel, C., Flammia, R. S., Horlemann, B., Sorce, G., Chierigo, F., Tian, Z., Saad, F., Graefen, M., Gallucci, M., Briganti, A., Terrone, C., Shariat, S. F., Tilki, D., Kluth, L. A., Mandel, P., Chun, F. K. H., Karakiewicz, P. I. (2022). Effect of chemotherapy in metastatic prostate cancer according to race/ethnicity groups Prostate, 82(6), 676-686 | Full text |
| 30 | Hawley, J. E., Pan, S., Kandadi, H., Chaimowitz, M. G., Sheikh, N., Drake, C. G. (2022). Analysis of Circulating Immune Biomarkers by Race in Men With Metastatic Castration-Resistant Prostate Cancer Treated With Sipuleucel-T Journal of the National Cancer Institute, 114(2), 314-317 | Full text |
| 31 | Giaquinto, A. N., Miller, K. D., Tossas, K. Y., Winn, R. A., Jemal, A., Siegel, R. L. (2022). Cancer statistics for African American/Black People 2022 CA Cancer Journal for Clinicians, 72(3), 202-229 | Full text |
| 32 | Farooq, M. Z., Shrivastava, T., Sarfraz, H., Ba Aqeel, S. H., Fu, P., Mangla, A. (2022). Racial disparity in survival of African-American (AA) patients diagnosed with metastatic prostate cancer: Analysis of surveillance epidemiology and end results program Journal of Clinical Oncology, 40(6 SUPPL) | Conference abstract/poster |
| 33 | Wurnschimmel, C., Wenzel, M., Colla Ruvolo, C., Nocera, L., Tian, Z., Saad, F., Briganti, A., Shariat, S. F., Mandel, P., Chun, F. K. H., Tilki, D., Graefen, M., Karakiewicz, P. I. (2021). Survival advantage of Asian metastatic prostate cancer patients treated with external beam radiotherapy over other races/ethnicities World journal of urology, 39(10), 3781-3787 | Full text |
| 34 | Khan, S., Chang, S. H., Hicks, V., Wang, M., Grubb, R. L., Drake, B. F. (2021). Improved survival with post-diagnostic metformin and statin use in a racially diverse cohort of US Veterans with advanced prostate cancer Prostate Cancer and Prostatic Diseases, doi: 10.1038/s41391-021-00475-5. Online ahead of print. | Full text |
| 35 | Gupta, S., Abbass, I. M., Craggs, C., Satram, S., My To, T., Mahrus, S., Sufan, R. I., Albarmawi, H. (2021). Overall survival of patients with metastatic castrate-resistant prostate cancer (mCRPC) who have PTEN tumor suppressor gene loss of function Journal of Clinical Oncology, 39(6 SUPPL) | Conference abstract/poster |
| 36 | Elmehrath, A. O., Afifi, A. M., Al-Husseini, M. J., Saad, A. M., Wilson, N., Shohdy, K. S., Pilie, P., Sonbol, M. B., Alhalabi, O. (2021). Causes of Death among Patients with Metastatic Prostate Cancer in the US from 2000 to 2016 JAMA Network Open, 4(8), e2119568 | Full text |
| 37 | Zhang, J., Liu, M., Pan, J., Wei, X. X., Harmon, M., Azzolina, J., Flanders, S. (2020). Real-world experience with sipuleucel-T (Sip-T) in Asian men with castrate-resistant prostate cancer (CRPC) Journal of Clinical Oncology, 38(15 SUPPL) | Conference abstract/poster |
| 38 | Muralidhar, V., Dee, E. C., Mahal, B. A., Wei, X. X., Sartor, O., Mouw, K. W., Nguyen, P. L. (2020). Association Between Black Race And Improved Survival Following Sipuleucel-T Immunotherapy In Metastatic Castrate-Resistant Prostate Cancer: Implications For Immune Biology And Integration Of Radiation Therapy With Immunotherapy International Journal of Radiation Oncology Biology Physics, 108(3 Supplement), e901 | Conference abstract/poster |
| 39 | Jogerst, K., Ali-Mucheru, M., Chang, Y., Pockaj, B., Stucky, C., Cronin, P., Wasif, N. (2020). Disparities in stage iv cancer outcomes: Treatment differences and association with overall survival Annals of Surgical Oncology, 27(Supplement 1), S93 | Conference abstract/poster |
| 40 | Deuker, M., Knipper, S., Pecoraro, A., Palumbo, C., Rosiello, G., Luzzago, S., Tian, Z., Saad, F., Chun, F., Karakiewicz, P. I. (2020). Prostate cancer characteristics and cancer-specific mortality of Native American patients Prostate Cancer and Prostatic Diseases, 23(2), 277-285 | Full text |
| 41 | Zhao, Feng, Wang, Jili, Chen, Meiqin, Chen, Danni, Ye, Sunyi, Li, Xinke, Chen, Xin, Ren, Guoping, Yan, Senxiang (2019). Sites of synchronous distant metastases and prognosis in prostate cancer patients with bone metastases at initial diagnosis: a population-based study of 16,643 patients Clinical and translational medicine, 8(1), 30 | Full text |
| 42 | Mazzone, E., Bandini, M., Preisser, F., Nazzani, S., Tian, Z., Abdollah, F., Soulieres, D., Graefen, M., Montorsi, F., Shariat, S., Saad, F., Briganti, A., Karakiewicz, P. I. (2019). The effect of race on survival after local therapy in metastatic prostate cancer patients Canadian Urological Association Journal, 13(6), 175-181 | Full text |
| 43 | Lim, J., Amantakul, A., Shariff, N., Lojanapiwat, B., Alip, A., Ong, T. A., Thevarajah, S., Ahmayuddin, F., Mathew, A., Sriplakich, S., Vuthiwong, J., Chong, F. L. T., Saad, M. (2019). Abiraterone in metastatic castrate resistant prostate cancer: A comparative analysis of the outcome and tolerance between Malaysian and Thai cohorts International Journal of Urology, 26(Supplement 2), 43-44 | Conference abstract/poster |
| 44 | Becker, D. J., Iyengar, A. D., Punekar, S. R., Jason, N. G., Zaman, A., Loeb, S., Becker, K. D., Makarov, D. (2019). Treatment of metastatic castration-resistant prostate cancer with abiraterone and enzalutamide despite PSA progression Anticancer Research, 39(5), 2467-2473 | Full text |
| 45 | Guo, X., Zhang, C., Guo, Q., Xu, Y., Feng, G., Li, L., Han, X., Lu, F., Ma, Y., Wang, X., Wang, G. (2018). The homogeneous and heterogeneous risk factors for the morbidity and prognosis of bone metastasis in patients with prostate cancer Cancer Management and Research, 10,1639-1646 | Full text |
| 46 | Ramalingam, S., Humeniuk, M. S., Hu, R., Rasmussen, J., Healy, P., Wu, Y., Harrison, M. R., Armstrong, A. J., George, D. J., Zhang, T. (2017). Prostate-specific antigen response in black and white patients treated with abiraterone acetate for metastatic castrate-resistant prostate cancer Urologic Oncology: Seminars and Original Investigations, 35(6), 418-424 | Full text |
| 47 | Moreira, D. M., Howard, L. E., Sourbeer, K. N., Amarasekara, H. S., Chow, L. C., Cockrell, D. C., Pratson, C. L., Hanyok, B. T., Aronson, W. J., Kane, C. J., Terris, M. K., Amling, C. L., Cooperberg, M. R., Freedland, S. J. (2017). Predicting Time From Metastasis to Overall Survival in Castration-Resistant Prostate Cancer: Results From SEARCH Clinical Genitourinary Cancer, 15(1), 60 | Full text |
| 48 | Rusthoven, C. G., Jones, B. L., Flaig, T. W., Crawford, E. D., Koshy, M., Sher, D. J., Mahmood, U., Chen, R. C., Chapin, B. F., Kavanagh, B. D., Pugh, T. J. (2016). Improved survival with prostate radiation in addition to androgen deprivation therapy for men with newly diagnosed metastatic prostate cancer Journal of Clinical Oncology, 34(24), 2835-2842 | Full text |
| 49 | Schmid, M., Ghani, K. R., Choueiri, T. K., Sood, A., Kapoor, V., Abdollah, F., Chun, F. K., Leow, J. J., Olugbade, K., Sammon, J. D., Menon, M., Kibel, A. S., Fisch, M., Nguyen, P. L., Trinh, Q. D. (2015). An evaluation of the 'weekend effect' in patients admitted with metastatic prostate cancer BJU International, 116(6), 911-919 | Full text |
| 50 | Sammon, J. D., McKay, R. R., Kim, S. P., Sood, A., Sukumar, S., Hayn, M. H., Hu, J. C., Kibel, A. S., Nguyen, P. L., Peabody, J. O., Saad, F., Sun, M., Varda, B., Menon, M., Choueiri, T. K., Trinh, Q. D. (2015). Burden of hospital admissions and utilization of hospice care in metastatic prostate cancer patients Urology, 85(2), 343-350 | Full text |
| 51 | Muralidhar, V., Mahal, B. A., Nguyen, P. L. (2015). Conditional cancer-specific mortality in T4, N1, or M1 prostate cancer: Implications for long-term prognosis Radiation Oncology, 10(1), 155 | Full text |
| 52 | Powell, I. J., Vigneau, F. D., Bock, C. H., Ruterbusch, J., Heilbrun, L. K. (2014). Reducing prostate cancer racial disparity: Evidence for aggressive early prostate cancer PSA testing of African American men Cancer Epidemiology Biomarkers and Prevention, 23(8), 1505-1511 | Full text |
| 53 | Taksler, G. B., Keating, N. L., Cutler, D. M. (2012). Explaining racial differences in prostate cancer mortality Cancer, 118(17), 4280-4289 | Full text |
| 54 | Tutrone, R., Pieczonka, C., Nordquist, L., Concepcion, R., Flanders, S., & Armstrong, A. (2019). Survival outcomes for metastatic castration-resistant prostate cancer with PSA less than 5.0 ng/ml treated with sipuleucel-t, overall and by race: data from the PROCEED registry. Society of Urologic Oncology - survival outcomes for metastatic castration-resistant prostate cancer with PSA less than 5.0 ng/ML treated with sipuleucel-T, overall and by Race: Data from the proceed registry. Retrieved April 19, 2022, from https://suo-abstracts.secure-platform.com/a/gallery/rounds/1/details/42 | Conference abstract/poster |

**Table A.5: Additional Studies**

| **Author, Year** | **Assessment of Race and Survival Conducted** | **Summary of Results** |
| --- | --- | --- |
| Lao, 2016 | Compared all-cause and cancer-specific survival between Maori and New Zealand European patients. | No statistically significance difference in survival was identified. |
| Siegel, 2020 | Presented 5-year survival rates for various racial/ethnic groups. | 5-year survival was highest among Asian/Pacific Islanders, followed by Hispanics, AI/AN, Black, and then White patients. |
| Sheean, 2022 | Assessed the association between myosteatosis at diagnosis and OS, with analyses conducted separately for Black and non-Black patients. | The presence of myosteatosis at diagnosis was associated with decreased OS, showing a more pronounced (but statistically nonsignificant) negative association in Black patients than in non-Black patients. |
| Hoeh, 2022 | Assessed the effect of chemotherapy on OS, with analyses conducted separately for various racial/ethnic groups. | The greatest OS benefit from chemotherapy was observed in White and Asian patients. No OS benefit from chemotherapy was observed in Black of Hispanic patients. |
| Giaquinto, 2022 | Overview of cancer statistics for African American/Black people in 2022. | A similar reduction in 5-year survival when prostate cancer is diagnosed at a distant stage was described for both Black and White patients. |
| Khan, 2021 | Compared the association between post-diagnostic statin use and OS, with analyses conducted separate for Black and White patients. | Post-diagnostic statin use was associated with improved OS in both Black and White patients. |
| Elmehrath, 2021 | Assessed differences in the cause of death, suicide rates, and explore mortality rates in various racial groups compared to the general US population. | For each of White, Black, Asian, or AI/AN, the risk of all causes of death, death from cancers other than prostate cancer^a^, and non-cancer causes of death is significantly greater than that of the general US population. White patients and Asian or Pacific Islander patients had an increased risk of suicide, but Black patients and American Indian or Alaska Native patients did not have an increased risk of suicide. |
| Lim, 2019 | Compared OS between Malaysian and Thai patients. | No statistically significance difference in survival was identified. |
| Muralidhar, 2015 | Compared conditional cancer specific mortality between White and non-White patients^b^ | “Among patients with M1 disease, conditional mortality was similar between white and non-white patients (data not shown)” |

^a^ Not significantly different from the general US population for AI/AN.
^b^ Non-White patients are not further characterized.
Abbreviations: AI/AN = American Indian/Alaska Native; OS = overall survival; US = United States

Table A.6: Risk of Bias Assessment of Cohort Full Text Publications

| **Author, Year** | **Selection** | **Comparability** | **Outcome** | **Total number of stars** |
| --- | --- | --- | --- | --- |
| Akinyemiju, 2018 | ★★★★ | ★★ | ★★ | 8 |
| Bernard, 2020 | ★★★★ | ★★ | ★★ | 8 |
| Bernard, 2017 | ★★★★ | ★★ | ★★★ | 9 |
| Borno, 2020 | ★★★★ | ★★ | ★★ | 8 |
| Deuker, 2021 | ★★★★ | ★★ | ★ | 7 |
| Garje, 2020 | ★★★★ | ★★ | ★★★ | 9 |
| He, 2021 | ★★★★ | ★★ | ★ | 7 |
| Keating, 2019 | ★★★★ | ★★ | ★★ | 8 |
| Lao, 2016 | ★★★ | ★★ | ★★ | 7 |
| Leuva, 2019 | ★★★ |  | ★★ | 5 |
| Marar, 2022 | ★★★★ | ★★ | ★★ | 8 |
| Parikh, 2017 | ★★★★ | ★★ | ★★ | 8 |
| Patel, 2018 | ★★★ | ★★ | ★★ | 7 |
| Sartor, 2020 | ★★★★ | ★★ | ★★★ | 9 |
| Siegel, 2020 | ★★★★ | ★ | ★★ | 7 |
| Smith, 2021 | ★★★ | ★★ | ★★ | 7 |
| Vengaloor Thomas, 2020 | ★★★★ | ★★ | ★★ | 8 |
| Weiner, 2020 | ★★★★ | ★★ | ★★ | 8 |
| Weiner, 2021 | ★★★★ | ★★ | ★★ | 8 |
| Wurnschimmel, 2021 | ★★★★ | ★★ | ★★★ | 9 |
| Zhang, 2021 | ★★★★ | ★★ | ★★ | 8 |
| Zhao, 2020 | ★★★ | ★★ | ★★ | 7 |
| Sheean, 2022 | ★★★★ | ★★ | ★★ | 8 |
| Oehrlein, 2022 | ★★★★ | ★★ | ★★ | 8 |
| Hoeh, 2022 | ★★★★ | ★★ | ★★ | 8 |
| Hawley, 2022 | ★★★ |  | ★ | 4 |
| Giaquinto, 2022 | ★★★★ | ★ | ★★ | 7 |
| Wurnschimmel, 2021 | ★★★★ | ★★ | ★★ | 8 |
| Khan, 2021 | ★★★ | ★★ | ★★ | 7 |
| George, 2021 | ★★★ | ★★ | ★★ | 7 |
| Elmehrath, 2021 | ★★★★ | ★★ | ★★ | 8 |
| Patel, 2020 | ★★★ | ★★ | ★★ | 7 |
| Lec, 2020 | ★★★★ | ★★ | ★★★ | 9 |
| Deuker, 2020 | ★★★★ | ★★ | ★★ | 8 |
| Zhao, 2019 | ★★★★ | ★★ | ★★ | 8 |
| Mazzone, 2019 | ★★★★ | ★★ | ★★ | 8 |
| Becker, 2019 | ★★★ | ★★ | ★★ | 7 |
| Guo, 2018 | ★★★★ | ★★ | ★★ | 8 |
| Moreira, 2017 | ★★★★ | ★★ | ★★ | 8 |
| Rusthoven, 2016 | ★★★★ | ★★ | ★★ | 8 |
| Schmid, 2015 | ★★★★ | ★★ | ★ | 7 |
| Sammon, 2015 | ★★★★ | ★★ | ★ | 7 |
| Muralidhar, 2015 | ★★★★ | ★★ | ★★ | 8 |
| Powell, 2014 | ★★★★ | ★ | ★★ | 7 |
| Taksler, 2012 | ★★★★ | ★★ | ★★ | 8 |
| Ng, 2021 | ★★★★ | ★ | ★★ | 7 |

Table A.7: Risk of Bias Assessment of Case Control Full Text Publications

| **Author, Year** | **Selection** | **Comparability** | **Exposure** | **Total number of stars** |
| --- | --- | --- | --- | --- |
| Ramalingam, 2017 | ★★★ | ★★ | ★★ | 7 |
